# Production of Fuel from Plastic Waste: A Feasible Business

**DOI:** 10.3390/polym13060915

**Published:** 2021-03-16

**Authors:** Irene Fahim, Omar Mohsen, Dina ElKayaly

**Affiliations:** 1 Department of Industrial Engineering, School of Engineering, SESC Research Center, Nile University, Nile Avenue, Giza 12655, Egypt; O.Mohsen@nu.edu.eg; 2 School of Business and Finance, New Giza University, Cairo 11435, Egypt; dina68@hotmail.com

**Keywords:** fuel, energy, plastic waste, recovery, calorific value, feasibility

## Abstract

This paper aims to conduct a feasibility study of producing fuel from plastic waste. It is a suggested approach to deal with the huge production of synthetic plastic around the world, so as to avoid its accumulation in landfills and the depletion of resources. Several types of research have addressed the conversion of plastic waste into energy, and in this study the authors focused on using pyrolysis to convert plastic to liquid oil. Accordingly, the volume of the waste was reduced significantly, and the produced liquid oil had a high calorific value in comparison to fossil fuel. The authors managed to develop a profitable business model for a facility producing fuel from plastic waste in Egypt. This project could be a very lucrative business opportunity for investors or venture capitalists interested in investing in green economy. A Business Model Canvas was used as a tool to identify how the different components of the business relate to each other.

## 1. Introduction

### 1.1. Plastic Waste: A Global Problem

Synthetic plastic production has reached 400 million tons worldwide. More than 50% of this figure is thrown in landfills or recycled. More than 15 million tons reach seas and oceans every year. There are several ways that plastic waste ends up in the ocean. Two-thirds of the waste is generated from land-based sources: litter left on the beach or washed down rivers and drains, and litter being dropped in towns and cities. The waste is also produced from industrial spills, badly managed landfill sites, by bins near the coast, or by rubbish being flushed down toilets. Most of these waste items are single-use plastics such as drink bottles, plastic bags, cotton bud sticks, sanitary items, and wet wipes. Incineration is also used to get rid of waste. However, U.S. emissions from plastic incineration reached 5.9 million metric tons of carbon dioxide in 2015, and they are expected to reach 49 million metric tons by 2030 and 91 million metric tons by 2050 (The Hidden Climate Polluter: Plastic Incineration—Global Alliance for Incinerator Alternatives, 2021). The burning of waste releases thousands of pollutants that affect people living near these incinerators. Furthermore, landfilling features a much lower climate impact than incineration. However, landfills are currently full, and there is no more space for waste accumulation. Landfilling contaminates soil and water, and also affects wildlife. Previously, the U.S. and other Western countries sent their contaminated waste to China, transferring the responsibility of waste management. In 2018, however, China closed its doors to the West’s contaminated recycling [1]. It has been concluded that waste plastic fuel has similar properties to diesel fuel and can be used instead of diesel [2].

### 1.2. Plastic Waste: A Severe Problem in Egypt

According to the plastics value chain mapping and assessment, “Egypt generates around 20 million tons of garbage and waste annually, with plastic waste assumed to represent 6% out of the total, distributed over Cairo (60%), Alexandria (16%), the Nile Delta (19%), and other regions including Upper Egypt, Suez Canal, and Sinai (5%). Out of the 970 kilotonnes of plastic waste generated annually, only a range of 30% is recycled, while 5% is reused, 33% is landfilled, and 32% is left to be burned”. The total amount of plastic waste represents 10% of all garbage in Egypt. The amount of plastic that is neither collected nor landfilled is 65%. This represents 1.3 million tons annually in Egypt, while Cairo only contributes 0.78 million tons. When burned, plastic waste releases harmful dioxins (highly toxic chemicals), which can be inhaled by humans and animals, deposited in soil and surface water, and deposited on plants. Uncollected plastics pose a threat to animals and sea life [3].

There are several successful waste management techniques that have been adopted in the Middle East. This research focuses on using plastic waste for fuel production. This process minimizes the volume of solid waste for landfills and would decrease CO_2_ emissions in Egypt caused by plastic waste by around 8% for the first year, and by 30% for the first five years. Moreover, it would decrease the high demand for fossil fuels products. Additionally, the carbon emissions produced using this novel fuel are 93% lower than those produced in the use of normal diesel and gasoline. Plastic wastes are subjected to a pyrolysis process using a catalyst [4]. The catalyst can be a blend of zeolite, clay, alumina, and silicates in various proportions [5]. There are three types of pyrolytic reactions differentiated by the processing time and temperature of the biomass: slow pyrolysis, fast pyrolysis, and flash pyrolysis [6]. The major byproducts of this process are char and gas. The proportion of the byproduct depends on temperature, heating rate, pressure, and residence time [7].

**Figure 1:**
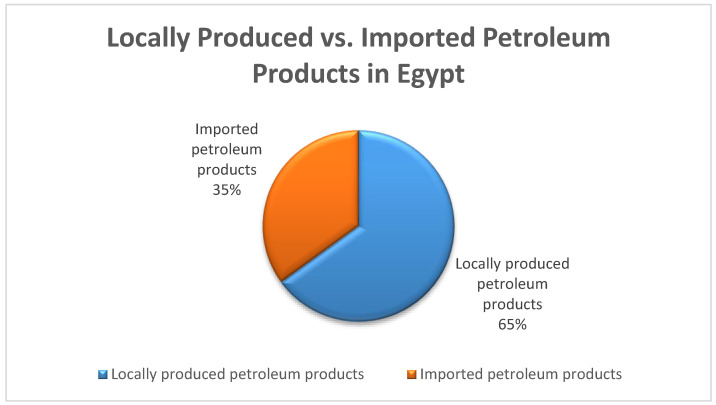
Local production and imports of petroleum products in Egypt.

Egypt has ten refineries which produce 760,000 barrels of petroleum products per day. The consumption of petroleum products in Egypt has reached 1.2 million barrels. Figure 1 shows that 35% of petroleum products in Egypt are imported, while 65% are locally produced. The cost of the imported oil reached around $7 billion in 2019 [8]. Thus, the fuel produced from plastic waste would fulfill the energy demands of the transport sector, and Egypt would have a continuous and sustainable source of fuel. Eventually, this could save a huge amount of money previously being spent on importing oil.

This study calculates the feasibility of combining several synthetic polymer matrix composites with different functions to produce a successful fuel processing system, using waste from high-density polyethylene (HDPE), polyethylene terephthalate (PET), polystyrene (PS), and polypropylene (PP). Getting rid of the waste of these poorly degradable synthetic polymers, which are undesirable due to their negative impact on health as well as on the environment, is successfully performed in this work by changing them to fuel through pyrolysis. The fuel industry would strongly benefit from this process as it would improve process sustainability. In this context, research into fuel and biorefineries will be immensely important in the near future. This work focuses on estimating the price of the development of fuel using a cost-effective process based on the valorization of synthetic plastic waste. In this context, the recycling of waste plastic as a fuel through pyrolysis in an inert atmosphere is an environmentally friendly solution.

## 2. Background on the Industrial Pyrolysis Process

This article focuses on calculating the cost of converting several types of plastic waste (PE, PS, PP, and PET) into fuel. The plastic waste is cleaned, dried, and size reduced. It is converted into liquid fuel using fast pyrolysis. HDPE is extracted from milk jugs, yogurt tubs, cleaning product containers, and body wash bottles. PET is collected from food and mouthwash containers. PP is found in plastic food storage containers, car parts, thermal vests, yogurt containers, and disposable diapers. PS is found in cups, insulation, packing materials, egg cartons, and disposable dinnerware [9]. The collected plastic is shredded and crushed into small pieces (1 × 3 cm^2^) to reduce the volume of the plastic in the reactor. The plastic pieces are washed to remove any toxic materials.

The catalyst needed is Zeolite Socony Mobil-5 (ZSM-5), which is a high-silica zeolite, widely used in the petroleum industry as a heterogeneous catalyst for hydrocarbon isomerization reactions. The chosen catalyst is a commercial catalyst. However, it must be dried in an oven to remove the moisture to below (5%) and further cracked to produce a much smaller particle size that will help in the reaction. The plastic-to-catalyst ratio is 10:1 [10]. The addition of the catalyst massively reduces the time needed for the process as well as the temperatures of the pyrolysis process, which results in an increase in the conversion rates for a wide range of polymers, which face significantly lower temperatures than they do during purely thermal pyrolysis. It also provides a high level of control in the distribution of the hydrocarbon products in LDPE, HDPE, PP, and PS pyrolysis [11].

### Synthesis of Liquid Fuels

**Figure 2:**
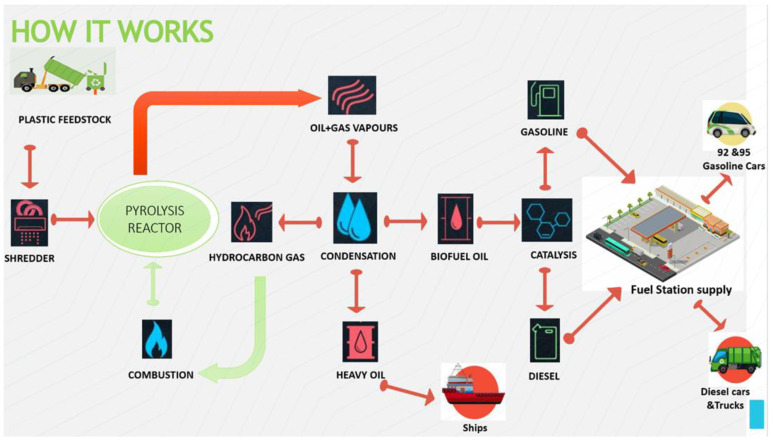
The pyrolysis process.

**Figure 3:**
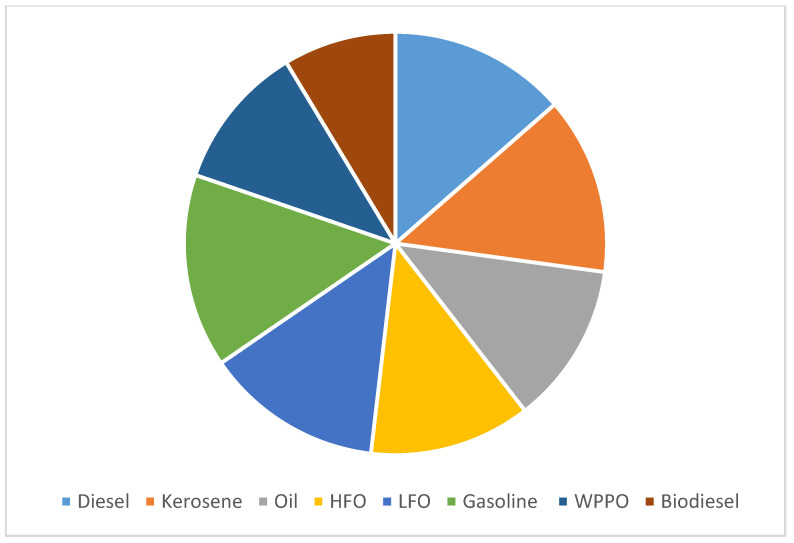
Similarity of plastic oil waste to the other fuel products [11].

The feedstock (plastic waste from different types of synthetic plastic) is shredded and mixed with the catalyst into the pyrolysis reactor with certain quantities and ratios, as illustrated in Table 1. The stainless-steel reactor is a fixed bed. The plastic is heated to reach a maximum temperature of 550 °C, at a heating rate of 15 °C/min. The products of the pyrolysis process are oil and gas vapor [12]. These products go through a condensation process to produce fuel oil, heavy oil, and light hydrocarbon gas [10], as shown in Figure 2. Hydrocarbons are stored and reused in the combustion process, so the energy in this system is self-sufficient. Biofuel oil is transported to refineries and converted to gasoline and diesel using the previously mentioned catalyst. Heavy oil is supplied to ships. The cost of this process is calculated to determine the economic feasibility of this process. One of the important properties of a fuel on which its efficiency is judged is its calorific value. The calorific value is defined as the energy produced when the unit mass of fuel is burned completely in sufficient air. Figure 2 shows a comparison between diesel, kerosene, furnace oil, heavy fuel oil (HFO), light fuel oil (LFO), gasoline, fuel from plastic waste (WPPO), and biodiesel. The calorific value of the fuel produced from plastic waste was estimated according to the IP 12/58 method. Its calorific value was 9829.3515 kcal/kg as shown in Figure 3, which is close to the calorific value of diesel [11,12,13].

## 3. Methodology

### 3.1. Feasibility of Producing Fuel from Plastic Waste

The statistics clearly demonstrated deficiencies in Egypt’s plastic waste management. Therefore, the authors decided to introduce a new business opportunity of establishing a production facility that converts 13,000 tons of plastic waste to fuel per day. This business concept is presented using a Business Model Canvas (BMC) as shown in Table 2. BMC helps to define how to get fuel products to their target customers by establishing a profitable business based on product innovation and efficient business processes, leading to a reduced risk of failure.

### 3.2. Calculating Cost Elements

To calculate the economic feasibility of converting plastic waste to fuel, the authors had to interview professional experts. In-depth interview was the selected data collection method. It was used to collect the needed detailed information. Around ten in-depth interviews were conducted and divided as per Table 3.

Based on these interviews, the authors identified both the investment and operational costs. The investment costs are the costs paid once during the establishment of the facility, as shown in Table 4.

The key assumptions for the feasibility study are identified in Table 5. The operational costs are the running costs paid monthly/annually as illustrated in Table 6. The technical expert suggested an initial daily active production capacity and we created several iterations until the authors agreed on the assumptions mentioned in Table 5. The authors also contacted a technical expert experienced in conducting technical feasibility studies to cross-validate the numbers.

### 3.3. Calculating the Revenue Projection

To project the revenues shown in Table 7, the authors contacted traders selling similar products. They estimated the price to be EGP 10,000–10,500. The annual increase is kept conservative, mainly covering the inflation rate.

### 3.4. Estimating the Profitability

The project is feasible based on the numbers presented in Table 8. A sensitivity analysis was also performed by increasing the cost by 10% and/or decreasing the price by 10%, and still the project remained feasible, as demonstrated in Table 9. However, the working capital remains a challenge, both to satisfy the short-term obligations and to achieve the projected profits.

## 4. Conclusions

This study demonstrated the importance of recycling plastic waste, especially in developing countries, as a means of saving unnecessary energy usage which in return decreases the production costs. Plastics can be easily converted into high-value fuel, which can be used as an alternative fuel. The volume of plastic waste present in the environment will be reduced along with the environmental effects such as excessive heating and greenhouse effects. The pyrolysis process is considered an effective, clean, and exceptionally successful technique in handling plastic solid waste, and it provides a cheap source of energy. Such a radical change should start with entrepreneurs suggesting new business ideas aimed at creating a wave leading to changing regulations and initiating governmental policies. Converting plastic waste into fuel in the Egyptian market would not only solve the plastic waste crisis in Egypt but would also decrease plastic pollution by avoiding incineration and landfilling, in addition to reducing the amount of imported oil barrels. Similarly, a company on Nova Scotia’s South Shore that plans to turn plastic waste into diesel and kerosene expects no significant environmental effects from its operations [14].Quantafuel is another Norwegian company collaborating with BASF Chemicals company to turn a global environmental problem into low-carbon products by collecting marine plastic waste and turning it into fuel [15].

## Figures and Tables

**Table 1:** Feedstock type and quantity [12].

Feedstock Type	Feedstock Quantity (kg)	Catalyst Quantity (kg)	Feedstock Ratio	Reaction Temp. (°C)	Heating Rate (°C/min)	Process Efficiency
HDPE	5	0.5	100%	550	15	94%
PET	5	0.5	100%	550	15	70%
PS	5	0.5	100%	550	15	80%
PP	5	0.5	100%	550	15	60%
PP/PET	5	0.5	50–50%	550	15	67%
PS/PP	5	0.5	50–50%	550	15	75%
Mixed	5	0.5	25% each	550	15	85.6–89.5%

**Table 2:** Business Model Canvas.

Business Model Canvas				
Key Partners ▪ Plastic producers ▪ Governmental agencies ▪ Financers ▪ Authorities concerned with the environment	Key Activities ▪ Recycling ▪ Assist in managing the plastic waste	Value Proposition ▪ Less pollution ▪ Better waste management system ▪ Lowering of the healthcare budget ▪ Business opportunity ▪ Better quality of life ▪ Job creation ▪ Energy saving	Customer Relations ▪ Consumer education/Awareness ▪ Good business environment	Customer Segments ▪ Manufacturers ▪ Associations
	Key Resources ▪ Recyclable materials ▪ Machinery ▪ Financial support		Distribution Channel ▪ Circular economy platforms ▪ Wholesalers	
Cost Structure ▪ Plant design and outlay ▪ R&D costs ▪ Raw materials and supplier selection		Revenue Stream ▪ Cost saving using recyclable materials flow ▪ Diversification (different types of plastic) ▪ Governmental support/grace period		

**Table 3:** Sample design of the experts’ interviews.

Covering	Number of Interviews	Segment (Who?)	Why?
Elements of the investment costs	1	Technical expert owning a company selling production lines and installing them	To find the investment cost to cover equipment costs. He was willing to customize the needed production line matching the required specifications, and he could identify the needed labor (blue collar).
	1	Chartered accountant	To find: ▪ The cost of establishing a company. ▪ The cost of issuing its legal documents. ▪ The annual increase needed to compensate the inflation rate. ▪ The currency conversion rate ▪ The norms of overheads, marketing, and other expenses ▪ The depreciation and taxation percentages
	2	Sales managers of office furniture companies (Mobica, Mohm Furniture, office furniture manufacturer in Egypt)	To find the cost of the office furniture
Elements of the operational costs	1	Salesperson in an industrial zone (Home—Polaris Parks)	To find the rental and utilities costs
	2	Human Resources managers of factories working in the same zone and related industries	To find: ▪ Suggested organization chart incorporating the blue collar workers and suggesting the suitable white collar workers ▪ Average salaries ▪ Other benefits
	2	Traders offering raw material who serve related industries	To find: ▪ The cost of the raw materials (plastic waste and necessary chemicals) ▪ Average selling price of fuel

**Table 4:** Investment costs.

Investment and Establishment Costs	Fees in EGP
Professional license fees	20,000
Equipment cost	2,000,000
Office furniture	50,000
Total fixed costs	2,070,000

Source: based on in-depth interviews conducted with experts—November 2020.

**Table 5:** Key assumptions for the feasibility model.

Element	Description	Source
Daily active production capacity	13,000 tons of fuel (out of 20,000 tons of plastic waste) The 20,000 tons was based on the amount of plastic waste collected daily. The production of 13,000 tons of fuel out of 20,000 tons of plastic waste was based on a preliminary experiment executed at lab scale	Calculated by the technical expert (one of the authors)
Number of working daysNumber of working months	26 days per month 12 months per year	Industry norm in Egypt that was confirmed by the technical expert
Monthly rental of a prebuilt factory	EGP 35,000 per month	Market price confirmed by in-depth interview with the sales manager of the industrial zone
Utilities and electricity	Electricity costs EGP 11,440 per month Utilities oil and water-repellent costs EGP 13,312 per month	
Price per ton of plastic waste	EGP 1500 per ton	Market price confirmed by in-depth interviews with traders
Labor	Total salaries (white- and blue-collar workers) = EGP 150,000 per month	Market price confirmed by in-depth interviews with HR individuals
Overheads, marketing, and other expenses	Overheads = 4% of Cost of Gods sold (COGS) Marketing expenses = 0.5% of COGS Other expenses = 0.5% of COGS Taxation = 22% of gross profit Depreciation = 20% of gross profit	Market norm confirmed by an in-depth interview with the chartered accountant

**Table 6:** Operational costs.

Category	Description	Operational Costs in EGP			
		Year 1	Year 2	Year 3	Year 4
Building and utilities	Monthly rentals	420,000	462,000	508,200	559,020
	Electricity	137,280	137,280	137,280	137,280
	Utilities, oil, and water repellent	159,744	159,744	159,744	159,744
Raw materials	Plastic waste + catalyst	9,360,000,000	10,296,000,000	11,325,600,000	12,458,160,000
Labor	Blue and white collar	1,800,000	1,800,000	1,800,000	1,800,000
Cost of goods sold	Sum of the abovementioned expenses	9,362,517,024	10,298,559,024	11,328,205,224	12,460,816,044
Overheads	Management and administrative expenses	374,500,681	411,942,361	453,128,209	498,432,641
Marketing	Marketing	46,812,585	51,492,795	56,641,026	62,304,080
Others	Exceptional expenses (might include insurance)	374,500,681	411,942,361	453,128,209	498,432,642
Total Operational Expenses		9,364,587,024	11,173,936,541	12,291,102,668	13,519,985,408

Source: based on in-depth interviews conducted with experts—November 2020.

**Table 7:** Revenue projection.

	Description	Revenue in EGP			
		Year 1	Year 2	Year 3	Year 4
Active production capacity in tons	13,000 tons will be produced every day (×26 days × 12 months)	4,056,000	4,461,600	4,907,760	5,398,536
% Sold		80%	85%	90%	95%
Price	Price per ton = EGP 10,200	10,200	10,200	10,200	10,200
Annual increase	Increase of selling price and costs mainly covering the inflation rate	10%	10%	10%	10%
Total Income		33,096,960,000	38,682,072,000	45,053,236,800	52,311,813,840

**Table 8:** Profitability.

Description	Revenue in EGP			
	Year 1	Year 2	Year 3	Year 4
Total income	33,096,960,000	38,682,072,000	45,053,236,800	52,311,813,840
Total costs	9,364,587,024	11,173,936,541	12,291,102,668	13,519,985,408
Gross profit	23,732,372,976	27,508,135,459	32,762,134,132	38,791,828,432
Taxation	1,186,618,648.80	1,375,406,773	1,638,106,706	1,939,591,421
Depreciation of equipment and office furniture	500,000	500,000	500,000	500,000.0
Net gains	22,545,654,327	26,132,628,686	31,123,927,425.36	36,852,137,010.6

**Table 9:** Sensitivity analysis.

Description	Total Gain in EGP			
	Year 1	Year 2	Year 3	Year 4
After decreasing the price to become EGP 1000	21,929,142,327	25,412,080,286	30,284,700,465	35,877,701,262
After increasing the total costs by 10%	21,656,018,560	25,071,104,715	29,956,272,671	35,567,738,396
After applying both changes	21,039,506,560	24,350,556,315	29,117,045,711	34,593,302,648

## Data Availability

The data presented in this study are available on request from the corresponding author.
